# The Antifibrosis Effects of Peroxisome Proliferator-Activated Receptor *δ* on Rat Corneal Wound Healing after Excimer Laser Keratectomy

**DOI:** 10.1155/2014/464935

**Published:** 2014-11-13

**Authors:** Yun Gu, Xuan Li, Tiangeng He, Zhixin Jiang, Peng Hao, Xin Tang

**Affiliations:** ^1^Clinical College of Ophthalmology, Tianjin Medical University, Tianjin Eye Hospital, Tianjin 300070, China; ^2^Tianjin Eye Hospital, Tianjin Eye Institute, Tianjin Key Laboratory of Ophthalmology and Vision Science, Nankai University of Eye Hospital, No. 4 Gansu Road, Heping District, Tianjin 300020, China; ^3^Department of Ophthalmology, Tianjin Medical University General Hospital, Tianjin 300052, China

## Abstract

Corneal stromal fibrosis characterized by myofibroblasts and abnormal extracellular matrix (ECM) is usually the result of inappropriate wound healing. The present study tested the hypothesis that the ligand activation of peroxisome proliferator-activated receptor (PPAR) *δ* had antifibrosis effects in a rat model of corneal damage. Adult Sprague-Dawley rats underwent bilateral phototherapeutic keratectomy (PTK). The eyes were randomized into four groups: PBS, GW501516 (a selective agonist of PPAR*δ*), GSK3787 (a selective antagonist of PPAR*δ*), or GW501516 combined with GSK3787. The agents were subconjunctivally administered twice a week until sacrifice. The cellular aspects of corneal wound healing were evaluated with in vivo confocal imaging and postmortem histology. A myofibroblast marker (*α*-smooth muscle actin) and ECM production (fibronectin, collagen type III and collagen type I) were examined by immunohistochemistry and RT-PCR. At the early stages of wound healing, GW501516 inhibited reepithelialization and promoted angiogenesis. During the remodeling phase of wound healing, GW501516 attenuated the activation and proliferation of keratocytes, which could be reversed by GSK3787. GW501516 decreased transdifferentiation from keratocytes into myofibroblasts, ECM synthesis, and corneal haze. These results demonstrate that GW501516 controls corneal fibrosis and suggest that PPAR*δ* may potentially serve as a therapeutic target for treating corneal scars.

## 1. Introduction

Corneal diseases are one of the leading causes of blindness in most developing countries [1]. Corneal trauma that involves the superficial penetration of the epithelium, Bowman's membrane, and anterior part of the stroma leads to tissue repair, which is often the onset of corneal fibrosis [2]. During the process of wound healing, epithelial cells, keratocytes, and inflammatory cells release a range of cytokines to stimulate epithelial regeneration, activate keratocytes, recruit immune cells, and deposit extracellular matrix (ECM) [3]. Typically, activated keratocytes migrate, proliferate, and differentiate into fibroblasts and *α*-smooth muscle actin (*α*-SMA)-positive myofibroblasts [4]. The changes in ECM composition and organization, which are characterized by collagen type III and proteoglycans low in keratan sulfate components [5], as well as less transparent myofibroblasts [4], contribute to corneal opacity. Successful treatments for corneal scarring are lacking. The commonly used steroids and mitomycin C (MMC) are associated with serious side effects [6, 7] while corneal transplantation is subject to the challenges of post-surgical complications and limited sources of high quality donors. Therefore, a new treatment that is both effective and safe needs to be explored.

Peroxisome proliferator-activated receptors (PPARs) are members of the nuclear receptor superfamily of ligand-activated transcription factors, including three PPAR isotypes: PPAR*α*, PPAR*β*/*δ* (called PPAR*δ* herein), and PPAR*γ*. The binding of the ligand induces PPARs to heterodimerize with a retinoid X receptor (RXR) and then bind to specific PPAR-responsive elements (PPREs) to regulate target gene expression. PPAR*δ* may also repress the transcription of some target genes by directly interacting with other transcription factors [8].

The PPARs are involved in a number of biological processes, including lipid metabolism, insulin sensitivity, inflammation, and cell proliferation and/or differentiation [9, 10]. While PPAR*γ* is widely being researched for its anti-inflammatory and antifibrosis activities in the liver [11], kidney [12], lung [13, 14], and eye [15], the potential functional roles of PPAR*δ* have emerged only in recent years. Extensive works by independent laboratories show that PPAR*δ* inhibits cell proliferation in keratinocytes [16], vascular smooth muscle cells (VSMC) [17], lung fibroblasts [18], and cardiac fibroblasts [19, 20]. The exposure of cardiac fibroblasts to PPAR*δ* agonists or the adenoviral overexpression of PPAR*δ* significantly decreases the *α*-SMA level, which indicates a reduced transdifferentiation from cardiac fibroblasts to cardiac myofibroblasts. Collagen synthesis is also decreased after the activation of PPAR*δ* in vascular smooth muscle cells [17] and cardiac fibroblasts [19, 20]. In vivo, PPAR*δ* agonist treatment protects against liver fibrosis by reducing inflammation [21]. Taken together, these findings suggest that PPAR*δ* activation may attenuate corneal fibrosis. The objective of this study was to investigate the roles of PPAR*δ* in corneal wound healing after laser ablation and to determine the antifibrosis effects of GW501516.

## 2. Materials and Methods

### 2.1. Animals and Surgery

Male adult Sprague-Dawley rats (240–260 g) were raised under conditions specified by the ARVO Resolution on the Use of Animals in Research. The experiments were performed with the ethics approval from the Tianjin Medical University Animal Ethics Committee.

For the detailed analysis, a precise wound (4.5 mm in diameter and 70 *μ*m in depth) was created with an excimer laser ablation protocol. The animals were anaesthetized with an intraperitoneal injection of 10% chloral hydrate at a dosage of 0.3 mL/100 g body weight. One drop of 0.5% proparacaine hydrochloride (Alcon-Couvreur, Puurs, Belgium) was applied topically to the eye prior to surgery. The rats were subjected to a bilateral regular phototherapeutic keratectomy (PTK) through the intact epithelium. The transepithelial PTK was performed centered over the pupil with a clinical 193 nm excimer laser (Star2, Visx Inc, Santa Clara, CA) by the same doctor.

### 2.2. Pharmacological Treatments

A total of 112 eyes were randomly divided into four groups, each consisting of 28 eyes: (1) laser-ablated corneas treated with 3 *μ*L of PBS as control, (2) laser-ablated corneas treated with 3 *μ*L of GW501516 (Santa Cruz, CA, USA) at a concentration of 1 mM, (3) laser-ablated corneas treated with 3 *μ*L of GSK3787 (Santa Cruz, CA, USA) at a concentration of 1 mM, and (4) laser-ablated corneas treated with 3 *μ*L GW501516 and GSK3787 at a concentration of 1 mM. Liquids were applied subconjunctivally immediately after PTK and twice a week until the rats were sacrificed. The other post-operative therapy consisted of topical 0.5% levofloxacin eye drops (Santen, Osaka, Japan) four times a day and ofloxacin eye ointment (Santen, Osaka, Japan) at night. Topical antibiotics were administered for one week. Neither non-steroid anti-inflammatory drugs nor steroids were used.

### 2.3. Ocular Surface Evaluation and Clinical Outcome Analysis

For slit-lamp biomicroscopy, the rats were anaesthetized as described above. A masked observer evaluated the ocular surfaces.

The areas of corneal epithelial defects were examined with fluorescein staining (0.1% sterile fluorescein solution in PBS) 0 h, 12 h, 24 h, 48 h, 72 h, and 7 days after wound generation. All results were recorded with a slit-lamp biomicroscope (SL-7F, Topcon, Tokyo, Japan) equipped with a digital camera. The areas of epithelial defects shown in photographs were then measured with the image analyzing software Image-Pro Plus, and the remaining corneal epithelial defect area (%) was calculated based on the initial corneal epithelial defect area ((remaining corneal epithelial defect area/initial corneal epithelial defect area) × 100) to evaluate the defect [22].

The levels of corneal opacity were graded with slit-lamp biomicroscopy one, two, three, and four weeks after PTK according to the grading standards set forth by Fantes (1990) [23]. The corneal clarity was graded as follows: grade 0, totally clear cornea with no opacity evident upon any method of microscopic slit-lamp examination; grade 0.5, trace or faint corneal haze seen only by indirect, broad tangential illumination; grade 1, haze of minimal density not easily observed with direct and diffuse examination; grade 2, mild haze easily visible with direct focal slit illumination; grade 3, moderate opacity that partially obscured the details of the iris; and grade 4, severe opacity that completely obscured the details of intraocular structures.

The degrees of corneal neovascularization (CNV) were quantified with slit-lamp biomicroscopy by taking photos of the cornea after mydriasis with 0.5% tropicamide and 0.5% phenylephrine mixed eye drops (Santen, Osaka, Japan). The wedge-shaped areas of vessel growth were then calculated in the photos using Image-Pro Plus with the following formula: *A* = *C*/12 × 3.1416 × [*r* ^2^ − (*r* − *l*)^2^], where *A* is the area, *C* is the number of clock hours of limbus involvement, *l* is the length of the longest neovascular pedicle from the limbus onto anterior cornea, and *r* is the radius of the cornea [24]. The degrees of CNV were compared among the groups using the ratio of the CNV area to the whole corneal area.

### 2.4. In Vivo Analysis of Corneal Haze with Corneal Confocal Microscopy

The rats were anaesthetized as described above for confocal microscopy in vivo using a Heidelberg Retina Tomograph III with Rostock Cornea Module (HRTIII-RCM, Heidelberg Engineering Inc., Germany) according to the manufacturer's instructions. Briefly, a drop of 0.2% carbomer gel (Bausch & Lomb Dr. Gerhard Mann Chem-Pharm, Berlin, Germany) was placed on the objective lens to provide immersion and prevent direct contact between the objective lens and the corneal surface. Centration was achieved using the central pupillary zone to maximize reproducibility. Subsequently, a series of images were collected to cover the entire stromal thickness with a continuous *z*-axis scan over the entire corneal stroma at 1 *μ*m increments starting from the basal layer of the corneal epithelium and ending at the corneal endothelium.

The average pixel intensity per plane was measured using Image-Pro Plus (Nippon Roper, Tokyo, Japan). A depth-intensity profile was generated from scans by plotting the average pixel intensity per plane as a function of corneal depth as previously described [25]. The total pixel intensity was calculated by measuring the area under the curve of interest. The average pixel intensity per micron was then measured based on the total pixel intensity divided by the thickness of interested stroma, which was measured by the axial distance.

### 2.5. Tissue Fixation, Sectioning, and Hematoxylin & Eosin (H & E) Staining

Four weeks after surgery, the rats were sacrificed and the eyes were enucleated. The obtained eyeballs were fixed in 10% neutral buffered formalin and embedded in paraffin wax. Central corneal sections (5 *μ*m thick) were cut parallel to the optical axis of the eye using an automated rotary microtome (Leica RM 2255, Leica Microsystems, Mannheim, Germany) and mounted on slides (Citotest Labware Manufacturing Co., Jiangsu, China). Standard Gill's II H & E staining was performed. The number of keratocytes was manually counted in five randomly selected nonoverlapping fields (×400) of the central cornea under microscope (Olympus BX50, Olympus, Tokyo, Japan) with a digital camera as previously described [26]. All H & E staining was performed at least three times to ensure that the results were consistent.

### 2.6. Tissue Fixation, Sectioning, and Immunofluorescent Staining

The rats were sacrificed two, three, and four weeks after surgery. The eyes were enucleated and embedded in liquid OCT compound (Sakura Finetek, Torrance, CA, USA). Frozen tissue blocks were stored in liquid nitrogen until sectioning. The central corneal sections (5 *μ*m thick) of the eyes were cut with a cryostat (Leica CM 1850, Leica Microsystems, Germany). The sections were placed on microscope slides and frozen at −80°C until staining was performed. Upon use, the slides were returned to room temperature, air-dried, and immersed in absolute methanol at −20°C for 2 min. The slides were then washed in PBST, permeabilized with 0.1% triton-X100 for 10 min, and blocked with 2% BSA for 30 min. The sections were then incubated with primary antibody overnight at 4°C. The primary antibodies used were peptide-affinity purified goat antibody against *α*-SMA (1 : 100 dilution; Abgent, USA), mouse monoclonal antibody against Col3a1 (1 : 100 dilution; Santa Cruz, CA, USA), and mouse monoclonal antibody against fibronectin (1 : 100 dilution; Santa Cruz, CA, USA). After PBST washing, the sections were incubated with FITC-labeled rabbit anti-goat IgG (H+L) (1 : 200 dilution; Earthox, USA) or TRITC-labeled rabbit anti-mouse IgG (H+L) (1 : 200 dilution; Earthox, USA) for 90 min at room temperature. The sections were then counterstained with DAPI (Vector Laboratories Inc., Burlingame, CA, USA) for 15 min and mounted in buffered glycerol (PH = 9.0) after PBST washing. For CD11b, the sections were not permeabilized, and PBS was used instead of PBST. Mouse monoclonal antibody against CD11b (2 *μ*g/*μ*L; Abcam, USA) was used as a primary antibody, and TRITC-labeled rabbit anti-mouse IgG(H+L) (1 : 200 dilution; Earthox, USA) was used as a secondary antibody. The sections were viewed and photographed using an inverted microscope (Nikon Inverted Microscope eclipse Ti-U, Nikon microscope, Tokyo, Japan) equipped with a Nikon digital camera and NIS-Elements Br Microscope Imaging Software. All immunofluorescent staining was performed at least three times to ensure that the results were consistent.

### 2.7. RT-PCR

The rats were killed four weeks after surgery. The eyes were enucleated, and the corneas were cut from the limbus with corneal scissors. The total RNA was extracted from the whole cornea using TRIzol (Invitrogen, Carlsbad, CA, USA) to assess the levels of fibronectin, collagen type I, collagen type III, and *α*-SMA. The RNA was extracted from the corneal stroma only and not the entire cornea to assess the levels of Ki67 antigen. The cDNA was generated using standard methods. Real-time PCR reactions were performed on a 96-well real-time PCR instrument (Mastercycler ep realplex, Eppendorf, USA) with the SYBR Premix Ex Taq (TaKaRa, Shiga, Japan) according to the manufacturer's protocol. The primer sequences were as follows: GAPDH: forward, 5′-CTCCCATTCTTCCACCTTTG-3′, reverse, 5′-ATGTAGGCCATGAGGTCCAC-3′; fibronectin: forward, 5′-CACTGGCCACACCTACAACC-3′, reverse, 5′-CTGGAATCATCTCTGTCAGC-3′; collagen type I: forward, 5′-TTGGGGCAAGACAGTCATCG-3′, reverse, 5′-TGTCCATTCCGAATTCCTGG-3′; collagen type III: forward, 5′-ATCAAACACGCAAGGCCATG-3′, reverse, 5′-AAGCAAACAGGGCCAATGTC-3′; Ki67 antigen: forward, 5′-TGGAGATCCAGATGTTAGGC-3′, reverse, 5′-TTGCATCTTTCTTGGCCCC-3′; *α*-SMA: forward, 5′-ATATTCTGTCTGGATCGGCG-3′, reverse, 5′-AGCATTTGCGGTGGACAATG-3′. The experiments were performed in triplicate and repeated a minimum of three times. The results were stated as the fold difference expression for each gene compared to that of GAPDH using the 2^−ΔΔ*Ct*^ method.

### 2.8. Statistics

All measurements were expressed as the mean ± SD except the data describing the CNV, which were expressed as the means. A Student's *t*-test was used to compare two groups, whereas an analysis of variance (ANOVA) was utilized for multiple comparisons for the epithelial defect area, relative intensities of reflectivity, mRNA level, and keratocytes count. Nonparametric Mann-Whitney and Kruskal-Wallis tests were used for the corneal haze and CNV comparisons. All statistical analyses were performed using SPSS (v15.0). *P* < 0.05 was considered statistically significant.

## 3. Results

### 3.1. Activation of PPAR*δ* Inhibited Corneal Epithelial Wound Healing

**Figure 1:**
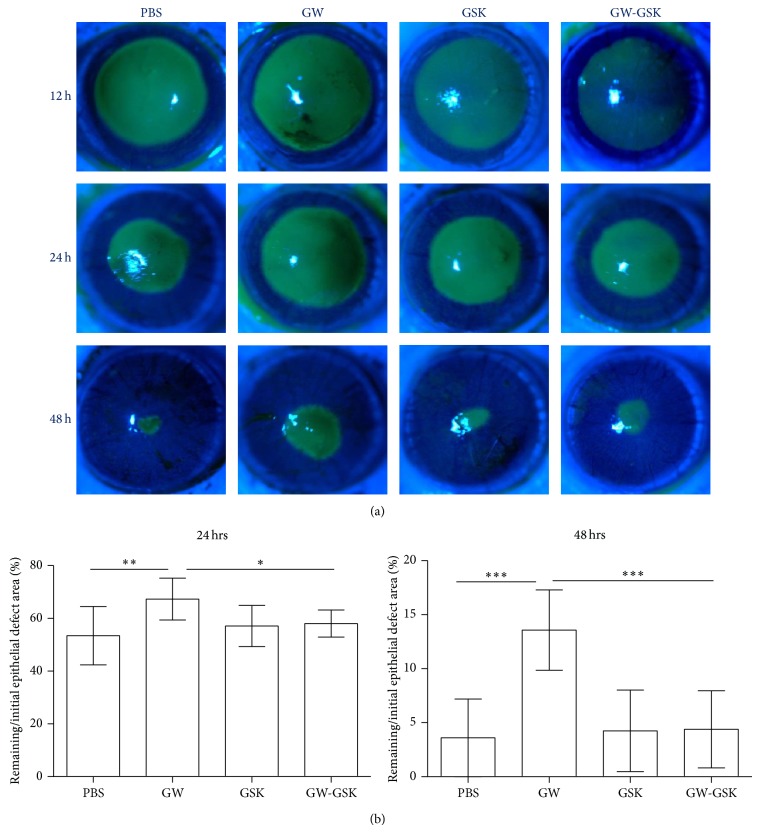
Subconjunctival injection of GW501516, a PPAR*δ* agonist, delayed reepithelialization during corneal wound healing in rats, which could be reversed by GSK3787, a PPAR*δ* antagonist. (a) Representative photographs of rats' ocular surfaces during the evaluation of corneal epithelial wound healing. Green areas represent fluorescein-stained areas of corneal epithelial defects. (b) The percentage rates of the remaining corneal epithelial defect area (% of each initial defect area) are shown at 24 and 48 h after surgery. The ranges of the *y*-axis differ in each graph. Data are presented as the mean ± SD (*n* = 7), and significant differences were statistically assessed by ANOVA. ^*^ *P* < 0.05, ^**^ *P* < 0.01, ^***^ *P* < 0.001 versus GW501516 group.

The effect of PPAR*δ* on corneal epithelial wound healing was assessed by measuring the epithelial defect area. The areas of epithelial defect generated by laser ablation immediately after surgery did not differ. As shown in Figure 1, the GW501516 group showed a significant delay in reepithelialization at 24 and 48 h after surgery (*P* < 0.05 to all other groups at the same time point). This group continued to show punctate or linear defects in the corneal epithelium at 72 h after ablation, which completely healed seven days after surgery.

### 3.2. Activation of PPAR*δ* Promoted Corneal Angiogenesis

**Figure 2:**
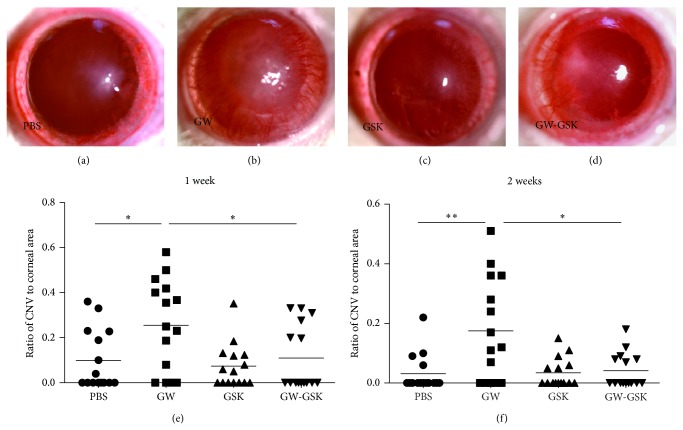
Subconjunctival injection of GW501516 promoted angiogenesis. Representative photographs of rats' ocular surfaces are shown at one week after treatment with PBS (a), GW501516 (b), GSK3787 (c), and GW501516 combined with GSK3787 (d). The degrees of CNV in the GW501516 group were significantly higher than all other groups at one week (e) and two weeks (f) after surgery. The ranges of the *y*-axis differ in each graph. The line represents the mean (*n* = 15), and differences were analyzed with a nonparametric test. ^*^ *P* < 0.05 versus GW501516 group.

The effect of PPAR*δ* on corneal angiogenesis was examined by measuring the area of CNV. One week after surgery, the CNV was most severe in GW501516-treated corneas, presenting with massive peripheral blood vessels (Figure 2(b)). Two weeks after surgery, the CNV markedly decreased in all groups, with some blood vessels reaching the central cornea. Subsequently, only a few small blood vessels were observed as blood vessels continued to vanish. Statistically, the CNV was the highest in the GW501516 group, with significant differences at one and two weeks after surgery (*P* < 0.05 to all other groups at the same time point) (Figures 2(e) and 2(f)). The CNV was low three and four weeks after surgery, and differences were not detected.

### 3.3. Effect of PPAR*δ* on Corneal Opacity

**Figure 3:**
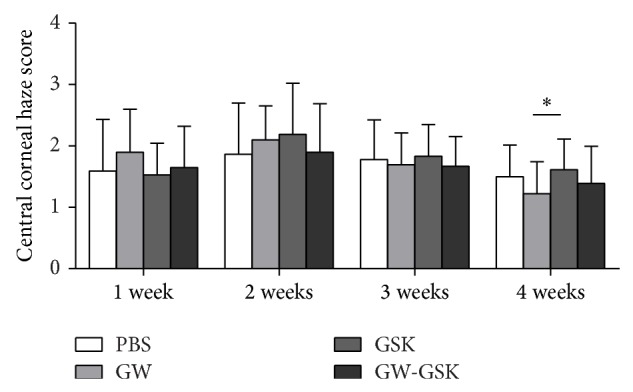
Central corneal haze scores are depicted in the bar graph. Four weeks after surgery, the haze score was lower in the GW501516 group than the GSK3787 group (*P* < 0.05); however, the GW501516 group did not significantly differ from the PBS group (*P* = 0.108). Data are presented as the mean ± SD (*n* = 18), and differences were analyzed with a nonparametric test. ^*^ *P* < 0.05 versus GW501516 group.

The corneal opacity was evaluated based on the central corneal haze score. One week after surgery, most of the corneas presented with corneal edema. The corneal haze became obvious two weeks after surgery. In some corneas, blood vessels reached the central cornea, which decreased the corneal transparency. Four weeks after surgery, the haze score was 1.50 ± 0.51 in the PBS group, 1.22 ± 0.52 in the GW501516 group, 1.61 ± 0.50 in the GSK3787 group, and 1.39 ± 0.61 in the GW501516 combined with GSK 3787 group. The haze score was lower in the GW501516 group than the GSK3787 group (*P* < 0.05); however, the GW501516 group did not significantly differ from the PBS group (*P* = 0.108) (Figure 3).

### 3.4. Effects of PPAR*δ* on the Activation and Proliferation of Keratocytes

**Figure 4:**
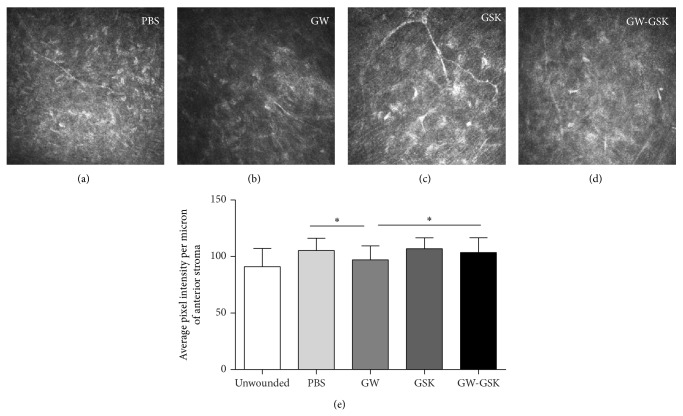
In vivo confocal micrographs of the anterior stroma of similar depth four weeks ((a)–(d)) after laser ablation. Corneas in the GW501516 group (b) showed quiescent keratocytes with low reflectivity, while the corneas in the GSK3787 group (c) showed active keratocytes with high reflectivity. (e) The relative intensities of reflectivity were evaluated based on the average pixel intensity per micron of the central anterior stroma. The data are presented as the mean ± SD (*n* = 4–6), and differences were analyzed by ANOVA. ^*^ *P* < 0.05, ^**^ *P* < 0.01 versus GW501516 group.

The cell morphology and ECM were observed using confocal microscopy with HRT III in vivo. The relative intensities of the reflectivity of the laser-ablated zone were then measured based on the average pixel intensity per micron of the anterior stroma. The anterior stroma was defined as the stroma from the epithelial-stromal interface to a depth of 30 microns, which was approximately one-third of the stromal thickness after laser treatment. Four weeks after surgery, the keratocytes in the GW501516 group (Figure 4(b)) were quiescent, and the reflectivities of the ECM were low. Moreover, the keratocytes in the GSK3787 group (Figure 4(c)) were active, and the reflectivities of the ECM were high. Correspondingly, the relative intensities of the reflectivity of the anterior stroma in the GW501516 group were the lowest, and this difference was significant (*P* < 0.05 to all other laser-ablated groups), which indicated that the corneal transparency was most improved in the GW501516 group (Figure 4(e)).

**Figure 5:**
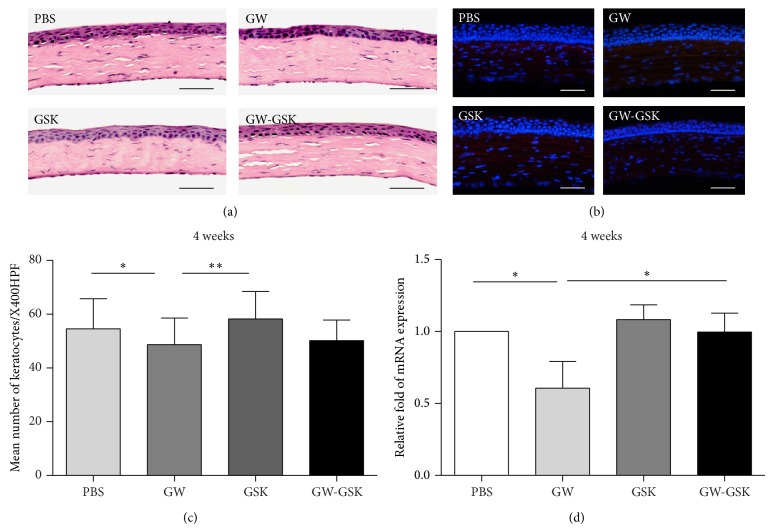
Effects of PPAR*δ* on keratocyte proliferation four weeks after surgery. (a) Representative images of H & E staining. Inflammatory or endothelial cells were not observed. Scale bars, 50 *μ*m. (b) Representative immunofluorescent staining images for CD11b. No positive cells were observed. DAPI-stained nuclei are shown in blue, and CD11b is shown in red. Scale bars, 50 *μ*m. Mean number of keratocytes (c) and mRNA expression of Ki67 antigen (d) are depicted in the bar graph. Data are presented as the mean ± SD (*n* = 3-4), and significant differences were statistically assessed using ANOVA. ^*^ *P* < 0.05, ^**^ *P* < 0.01 versus GW501516 group.

The cellular aspects of corneal wound healing were evaluated by H & E staining and immunofluorescent staining. Four weeks after surgery, the epithelium was stratified in all groups. Inflammatory or endothelial cells were not observed in the central corneal stroma in any group based on H & E staining (Figure 5(a)). The immunofluorescent staining for CD11b was negative in all groups (Figure 5(b)), suggesting the absence of neutrophils and macrophages. Based on the findings mentioned above, the number of keratocytes and mRNA level of Ki67 antigen were examined to elucidate the effects of PPAR*δ* on the cell proliferation of keratocytes. In the GW501516 group, the mean number of keratocytes was significantly lower than in the PBS and GSK3787 groups (in both *P* < 0.05) (Figure 5(c)). The mRNA level of Ki67 antigen was the lowest in the GW501516 group (*P* < 0.05 to all other groups) (Figure 5(d)).

### 3.5. Effects of PPAR*δ* on the Transdifferentiation of Keratocytes into Myofibroblasts and ECM Synthesis

Myofibroblasts express *α*-SMA, and increased secretions of fibronectin and collagen are key hallmarks of myofibroblast differentiation. The levels of *α*-SMA, fibronectin, and collagen type III protein were examined by immunofluorescent staining, and the mRNA levels of *α*-SMA, fibronectin, collagen type III, and collagen type I were measured by RT-PCR.

**Figure 6:**
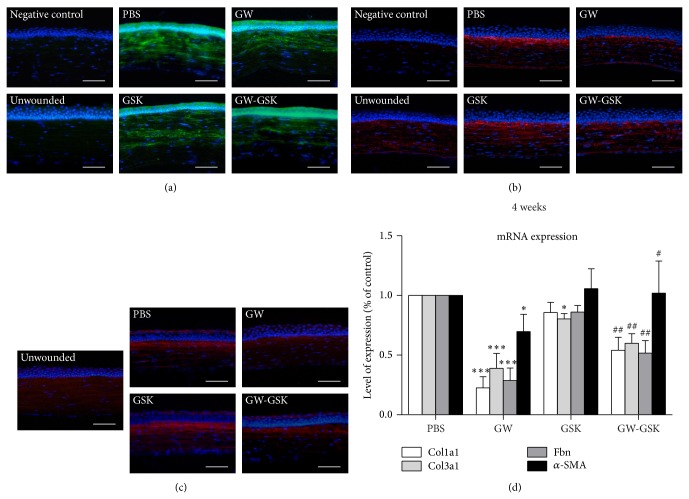
Representative immunofluorescent staining images for *α*-SMA (a) two weeks after laser treatment and fibronectin (b) and collagen type III (c) four weeks after laser treatment. DAPI-stained nuclei are shown in blue, *α*-SMA is shown in green, and fibronectin and collagen type III are shown in red. Scale bars, 50 *μ*m. (d) mRNA expression of *α*-SMA, fibronectin, collagen type III, and collagen type I at four weeks is depicted in the bar graph. Data are presented as the mean ± SD (*n* = 3-4), and significant differences were analyzed by ANOVA. ^*^ *P* < 0.05, ^**^ *P* < 0.01, ^***^ *P* < 0.001 versus PBS group. ^#^ *P* < 0.05, ^##^ *P* < 0.01, ^###^ *P* < 0.001 versus GW501516 group.

*α*-SMA-positive cells were absent in the stroma of the unwounded cornea based on immunofluorescent staining. Two weeks after laser treatment, *α*-SMA-positive cells were observed in the anterior and midstroma in all groups (Figure 6(a)). Significant differences were not detected among groups. Three and four weeks after laser treatment, *α*-SMA-positive stromal cells were seldom observed in the posterior stroma. Four weeks after laser treatment, GW501516 significantly reduced the mRNA level of *α*-SMA compared to all other groups (*P* < 0.05 to all other groups) (Figure 6(d)).

In the unwounded cornea, immunofluorescent staining for fibronectin was evident in the basement membrane, stroma, and Descemet's membrane. Four weeks after laser treatment, fibronectin was mainly expressed in the anterior stroma of the central cornea in all groups. The expression was distinct and consistent along the laser-injured site. We observed that fibronectin expression was lower in the GW501516 group than in the PBS and GSK3787 groups (Figure 6(b)). The mRNA level of fibronectin (Figure 6(d)) was the lowest in the GW501516 group (*P* < 0.01 compared to all other groups). Collagen type III was not detected in the unwounded cornea. Four weeks after laser treatment, the expression of collagen type III was lower in the GW501516 group than in the PBS and GSK3787 groups (Figure 6(c)). The mRNA level of collagen type III (Figure 6(d)) was the lowest in the GW501516 group (*P* < 0.01 compared to all other groups). Similarly, the mRNA level of collagen type I was the lowest in the GW501516 group (*P* < 0.01 compared to all other groups).

## 4. Discussion

The present study focuses on a novel examination of the roles of PPAR*δ* in corneal wound healing after laser ablation. GW501516 was found to inhibit the reepithelialization of corneal wounds that involve the anterior stroma. The ligand activation of PPAR*δ* was proangiogenic in the wounded cornea, which was not explored before. We demonstrated that PPAR*δ* attenuated the corneal opacity during the remodeling phase of wound healing. Corneal confocal microscopy was utilized to demonstrate for the first time that PPAR*δ* inhibited keratocyte activation in vivo. Furthermore, the study supported that the activation of PPAR*δ* attenuated the proliferation and transformation of keratocytes as well as ECM synthesis. These findings are consistent with previous studies of PPAR*δ* reporting the potent antifibrosis effects on several different types of cells and tissues [13, 17–21, 27].

In this study, we demonstrated that GW501516 delayed the reepithelialization of corneas in which the anterior stroma was injured in a PPAR*δ*-dependent manner. PPAR*δ* reportedly attenuated the proliferation of keratinocytes in skin [16]. In the cornea, the role of PPAR*δ* in epithelial wound healing was first demonstrated by Yoshikuni, who showed that it promoted the reepithelialization process via an antiapoptotic effect [22]. In Yoshikuni's study, the topical administrations of GW501516 accelerated epithelial wound healing in the mechanically ablated cornea of rabbits with an intact anterior stroma in vivo and corneal epithelial wound closure by human corneal epithelial cells in vitro. Our results, which were obtained with a different model, contradict Yoshikuni's study. In an animal model with a larger wound size (4.5 mm versus 3.0 mm in diameter) generated with a laser, the epithelial cells were subjected to microenvironments that were different from the ones in Yoshikuni's study. Moreover, a damaged anterior stroma complicated the reepithelialization process because of epithelial-mesenchymal interactions. Similar to the application of MMC after laser treatment [7], GW501516 delayed reepithelialization without increasing corneal haze in the long term, suggesting that the activation of PPAR*δ* inhibited the activation of keratocytes. Further research is needed to clarify the effect of PPAR*δ* on corneal epithelial cells under different conditions.

In recent years, many studies have confirmed that the activation of previously quiescent keratocytes and the generation of myofibroblasts are responsible for ECM deposition [2, 3]. Following injury to the corneal stroma, keratocytes undergo a sequence of morphological and functional changes [28]. Briefly, the keratocytes located at the wound bed undergo apoptosis hours after injury [29], resulting in an acellular wound bed. The adjacent keratocytes are then activated, begin to proliferate, and migrate toward the damaged area. When they reach the wound bed, they take on a repair phenotype with a fusiform shape, multiple nucleoli, and lack of cytoplasmic granules, like typical fibroblasts. As wound healing progresses, a subset of repair fibroblasts may transform into myofibroblasts. The *α*-SMA-positive myofibroblasts usually take on a stellate shape and are larger than the fibroblasts. Thus, the cell morphology can be used to distinguish the cell phenotype. In our study, the differences in cell morphology observed by in vivo confocal microscopy among all groups four weeks after surgery demonstrated that the ligand activation of PPAR*δ* inhibited the activation of keratocytes. The quantitation of corneal confocal images, keratocyte counts, and Ki67 antigen mRNA levels all indicated that GW501516 decreased the proliferation of keratocytes, which was consistent with the inhibition effects of PPAR*δ* on the activation of keratocytes. The excimer laser ablation is known as an excellent way of inducing reproducible corneal wounds with defined shape, size, and depth to generate a band of myofibroblasts in the subepithelial stroma of rats, rabbits, and humans [30]. In our study, the bands of myofibroblasts were confirmed two weeks after surgery via immunofluorescent staining for *α*-SMA. Four weeks after surgery, the lower mRNA level of *α*-SMA implied decreased myofibroblasts differentiation, although *α*-SMA-positive cells were seldom detected. In agreement with these changes, the decreased synthesis of collagen and other components of the ECM also indicated the reduced cell function of myofibroblasts and keratocytes. All of these data supported the antifibrosis effects of GW501516 in a PPAR*δ*-dependent manner. The haze score did not statistically differ between the GW501516 group and the PBS group. This lack of difference may be due to the modesty of the antifibrosis effect of PPAR*δ* and insufficient sample size to detect the small difference.

In our study, we demonstrated that the activation of PPAR*δ* inhibited the proliferation and transdifferentiation of keratocytes and associated ECM synthesis. These findings are consistent with reports of PPAR*δ* from cardiac fibroblast [19, 20] and arterial smooth muscle cells [17]. Several studies confirm that the antiproliferation effect of PPAR*δ* is associated with the upregulation of the PPAR-responsive cell cycle inhibitory G0S2 gene, while PPAR*δ* diminished the fibroblast-to-myofibroblast transdifferentiation possibly via increased levels of PTEN expression [17, 19]. Our in vitro experiment examined the effects of PPAR*δ* on the migration, proliferation, and cell cycle regulation gene expression in TGF*β*1-stimulated keratocytes. In vivo, the antifibrosis effect of PPAR*δ* may involve other mechanisms, including an anti-inflammatory effect. Indeed, PPAR*δ* is already known to play a role in inflammatory control [31]. GW0742, a synthetic high-affinity PPAR*δ* agonist, reportedly halts inflammatory and apoptotic processes induced by bleomycin [27]. In this study, we used excimer laser ablations to produce corneal wounds. The immune reaction after laser treatment is often most obvious within the first three days, and most studies focused on the time within one week after laser treatment. In our study, immunofluorescent staining at four weeks did not identify CD11b-positive cells, suggesting the absence of an immune reaction at this time. However, investigating the possible contribution of GW501516 to inhibit immune cells at early time points remains important because early immune reactions are an inseparable part of the wound healing process. Similarly, the effects of GW501516 on apoptosis should be investigated because myofibroblasts are believed to disappear via apoptosis during the late phases of wound healing.

To the best of our knowledge, our study is the first to implicate PPAR*δ* in the promotion of angiogenesis in corneal wound healing. Although the effect of PPAR*δ* on angiogenesis remains unclear, a considerable body of evidence supports its proangiogenic properties. Studies have shown that the activation of PPAR*δ* increases endothelial cell proliferation and function both in vitro and in vivo [32, 33]. In another study, GW0742 caused a significant and dose-responsive increase in tube formation, indicating that PPAR*δ* may regulate angiogenesis via a prodifferentiation/maturation mechanism [34]. Despite the controversy over how PPAR*δ* promotes angiogenesis, PPAR*δ* is widely accepted to be proangiogenic, which agrees with our results. Angiogenesis is undesirable in the cornea because the newly formed blood vessels result in not only the loss of corneal transparency but also the loss of immunological privilege of the cornea. The associated angiogenesis may alter the wound healing process and possibly the immune reaction of cornea. Overall, the mechanism by which PPAR*δ* promotes angiogenesis needs to be elucidated to avoid the unfavorable side effects.

Taken together, our data demonstrated that GW501516 delayed corneal reepithelialization, promoted CNV, and attenuated corneal fibrosis, when administrated immediately after laser ablation. As the undesired effects took place mainly at early stages, we hypothesized that starting GW501516 treatment at a later time point might avoid detrimental early effects while maintaining the beneficial late effects. It may be a useful strategy to start treatment somewhere between day 3 and day 7 after wound generation, and we will examine it in future research.

## 5. Conclusions

We reported for the first time that the ligand activation of PPAR*δ* inhibited the activation and proliferation of keratocytes. The transdifferentiation from keratocytes to myofibroblasts and ECM synthesis were also reduced. Thus, PPAR*δ* has potent antifibrosis effects on corneal wound healing, but the target should be carefully chosen because it delays corneal reepithelialization and also promotes CNV.

## Supplementary Material

There was a flow diagram of enucleated eyes in the Supplementary Material. Additionally, the effects of PPAR*δ* agonist/antagonist on normal corneal surface and circulating leukocyte counts were demonstrated.
