# Supplementary material for: The Antifibrosis Effects of Peroxisome Proliferator-Activated Receptor δ on Rat Corneal Wound Healing after Excimer Laser Keratectomy
Source: PPAR Res. 2014 Nov 13;2014:464935. doi: 10.1155/2014/464935 (PMC4248330; doi:10.1155/2014/464935)
Supplement: Supplementary file 1 — There was a flow diagram of enucleated eyes in the Supplementary Material. Additionally, the effects of PPARδ agonist/antagonist on normal corneal surface and circulating leukocyte counts were demonstrated. [file 464935.f1.pdf]

Supplementary material

Part 1. The flow diagram of enucleated eyes

Enucleated eyes were sorted into different outcome measurements. Here is the flow diagram of enucleated eyes:

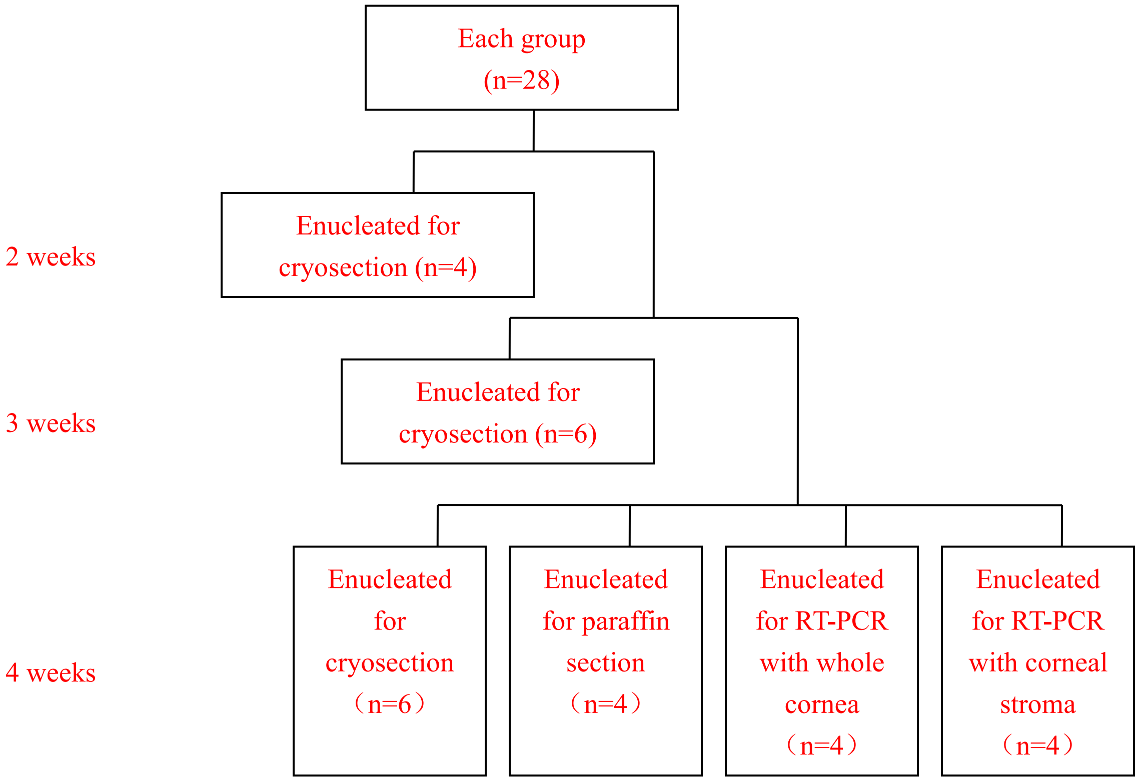

Part 2. Effects of PPARδ agonist/antagonist on normal corneal surface

Materials and methods:

12 rats with unwounded eyes were randomly divided into four groups, each consisting of six eyes: (1) unwounded corneas treated with 3 μl of PBS, (2) unwounded corneas treated with 3 μl of GW501516 (Santa Cruz, CA, USA) at a concentration of 1 mM, (3) unwounded corneas treated with 3 μl of GSK3787 (Santa Cruz, CA, USA) at a concentration of 1 mM and (4) unwounded corneas treated with 3 μl GW501516 and GSK3787 at a concentration of 1 mM. Liquids were subconjunctivally applied twice per week for four weeks.

Result:

No haze or corneal neovascularization was found in the uninjured corneas. H & E staining of the tissue showed no histological change. The data demonstrated that a subconjunctival injection of PPARδ agonist/antagonist did not harm the normal cornea.

Part 3. Effects of PPARδ agonist/antagonist on circulating leukocyte counts

Materials and methods:

The blood samples (2 ml for each rat) were collected from the rats' orbital venous plexus

immediately before sacrifice. The complete blood counts were performed using an automated hematology analyzer (Sysmex SE-9000™, TOA, Japan).

Result:

As shown in Table 1, differences were not detected between any of the groups.

Table 1. WBC counts in peripheral blood after subconjunctival injection of PPARδ agonist/antagonist for four weeks

	n	WBC counts（$\times 10^9$/L）
unwounded corneas + PBS group	3	11.26 ± 2.65
unwounded corneas + GW501516 group	3	11.15 ± 1.78
unwounded corneas + GSK3787 group	3	9.35 ± 3.69
unwounded corneas + GW-GSK group	3	11.27 ± 2.65
laser ablated corneas + PBS group	3	9.14 ± 2.66
laser ablated corneas + GW501516 group	3	10.33 ± 2.68
laser ablated corneas + GSK3787 group	3	10.58 ± 1.99
laser ablated corneas + GW-GSK group	3	9.95 ± 3.65
